# IPL: Image-Assisted Person Localization for Underground Coal Mines

**DOI:** 10.3390/s18113679

**Published:** 2018-10-29

**Authors:** Qiang Niu, Xu Yang, Yuqing Yin

**Affiliations:** 1 School of Computer Science and Technology, China University of Mining and Technology, Xuzhou 221000, China; niuq@cumt.edu.cn (Q.N.); yinyuqing@cumt.edu.cn (Y.Y.); 2 Mine Digitization Engineering Research Center of the Ministry of Education, Xuzhou 221000, China

**Keywords:** localization, coal mine, step information, image recognition, base station

## Abstract

Underground personnel localization is highly important in the operations of coal mines. Considering the special underground environment, this paper introduces a novel localization scheme based on step detection and image recognition technologies, which makes use of unique characteristics of the underground environment like the dark environment and the miner’s lamp. Since the underground topology is relatively simple, the miner can be located only by step information. However, the localization with step information always causes the problem of cumulative error. To solve this problem, we rebuild a special base station with a camera in a dark underground environment. A miner’s lamp, which every miner carries, can simply transform to irradiate unique shapes (such as triangles, rectangles, and circles) and every coal miner at the base station can identify these shapes based on image recognition technologies. Thus, we can obtain the miner’s precise position when he/she is passing by a base station. In that way, we can correct the localization results to solve cumulative error. We implemented our algorithm in indoor and underground environments. The experimental results show that 96% of spatial errors were 2.5 m or less.

## 1. Introduction

Underground personnel real-time positions are important for relief efforts when a mine accident occurs [1]. The aim of person localization in coal mines is to find the stranded miners rapidly and ensure the safety of miners when the accident happens. Although the underground personnel real-time position plays an important role in coal mine production, itself is a challenging problem due to the complex environment of coal mine [2].

Underground localization in coal mine can be regarded as one kind of special indoor localization [3]. Many localization solutions are dedicated for person localization systems. In view of whether device is required, there are two basic types of methods:1) device-based, and device-free. Device-based indoor localization systems can provide position service to the target who wears a specific smartphone-based [4] or tag-based [5] device. These approaches are broadly used to support numerous indoor location-based services(ILBSs). In pace with the recent proliferation of smartphones for social communications, smartphone-based indoor localization has become popular in providing various ILBSs. Device-free indoor localization approaches aim to mitigate the necessity of carrying devices in order to apply for special applications such as intrusion detection, tracking identify and safety precaution for elders. However, for underground special environment, there exist the severe conditions of signal propagation (multipath and NLOS) and rough sidewall surfaces [6,7], which make previous solutions ineffective.

In this paper, we propose an image-assisted person localization (IPL) algorithm for underground coal mine. This algorithm is built for a special underground coal mine environment. The mine construction is very simple and has a generally long and narrow roadway [8]. Thus, we can utilize step detection to localize humans. However, it is generally known that step-based localization algorithm has a cumulative error problem. To solve this problem, we need dense base stations placed in correct positions. Thus, the research question is how to establish a low-cost and reliable base station in a mine.

The establishment of the base station makes full use of the dark environment and the miners’ lamp. Considering that every coal miner has a lamp, we simply transform the miner’ s lamp to make it irradiate unique shapes (such as triangles, rectangles, and circles). The base station utilizes a camera that photographs the unique shapes, and then image recognition technology is used to identify these shapes. Thus, coal miners can be accurately located whenever they pass by the base station, thereby solving the cumulative error problem of a step-based localization algorithm.

Our main idea is to use a low-cost accelerator to obtain the step information and calculate the stride-length. Thus, we use the step and stride-length to locate the coal miner. This localization method is not accurate due to the cumulative error problem. Thus, we further design a low-cost base station to correct the localization results based on image recognition technology. The proposed localization scheme consists of four phases: (1) construction of base station, (2) detection of step, (3) calculation of stride-length, and (4) real-time localization. Specifically, our key contributions in this paper are as follows:

- To the best of our knowledge, this is a novel image-assisted step-based human localization scheme for mines, which uses the step detection and image recognition techniques. It can achieve better positioning accuracy with low-cost.
- We design a novel image-assisted method to improve the cumulative error problem of step-based localization scheme. Humans can be accurately located when they pass by the base station.
- The performance of the proposed design is evaluated in indoor and underground environments. The results verify the effectiveness of the localization scheme.

The remainder of this article is organized as follows: The next part describes the related works on localization schemes. Part 3 presents the main design with the detailed algorithm analysis. Part 4 evaluates the algorithm in indoor environment and underground environment. Finally, Part 5 concludes the paper and provides future work directions.

## 2. Related Work

Underground localization can be regarded as a special indoor localization. Many indoor localization schemes have previously been proposed, and they can be categorized into two classes: (1) device-based indoor localization and (2) device-free indoor localization.

Device-based localization requires direct communication between the node and the base station, and it transforms the received signal strength, arrival time difference, or arrival angle to distance [9,10,11]. Then, the coordinates of the target are estimated according to the geometric relationship or fingerprint library matching. These localization solutions can be classified into two categories: smartphone-based and tag-based. It is well-known that smartphones has powerful storage capacity and easy hackability. Furthermore, numerous kinds of modalities (e.g., WiFi [12,13], FM radio [14,15], Bluetooth, microphone, etc.) which are embedded in smartphones nowadays can be utilized to realize localization purposes separately or integrally. On this occasion, extensive approaches which are based on smartphone are springing up to deal with the problem of indoor localization.At the same time, a large amount of candidates appear to take the place of those require specific hardware for achieving the same goal, like infrared [16,17], ultrasonic, RFID [18,19,20] and Zigbee [21], and they are classified based on tag. However, these methods’ need for hardware leads to higher costs and causes difficulty in directly applying the hardware to underground coal mine localization.

In spite of the considerable progress of improving localization performance which are made by device-based techniques, when associated with the above crucial applications device-free techniques are more adorable [22]. Safety precaution protects disabled individuals or lonely elders from apoplexia, fall, empyrosis, and so on. Intrusion detection and tracking recognize if there are anomaly objects existing and acquire their locations in an area of interest. Border protection prohibits terrorists from entering a restricted zone. On these occasions, it is in urgent need of a cost-effective and suitable tool for device-free indoor localization. In recent years, device-free localization has become a research hotspot. According to the different hardware, it can be divided into video-based [23], UWB-based [24], wireless sensor-based [25], and passive localization based on Wi-Fi [26]. The cost of deploying video-based and UWB-based localization systems on a large scale is high. In addition, for the special underground coal mine environment device-free localization algorithms have a low positioning accuracy due to the signal reception intensity is restricted by localization accuracy because it cannot achieve multi-target motion tracking.

Currently, the research on underground coal mine localization is combined with unique characteristics of coal mine communication, which introduces the localization system on the ground to the underground, and then uses related ranging technology to recognize positions and track moving targets, such as personnel and equipment underground. Reference [27] discusses a type of anchor-free localization algorithm for target tracking in underground wireless sensor networks. However, the anchor-free localization algorithm only makes use of the multidimensional scaling and sorting of information to complete the positioning and the precision of this algorithm is difficult to improve. Reference [28] proposed a study of a coal-mine underground positioning algorithm based on kernel function and particle filter.

## 3. System Design

**Figure 1:**
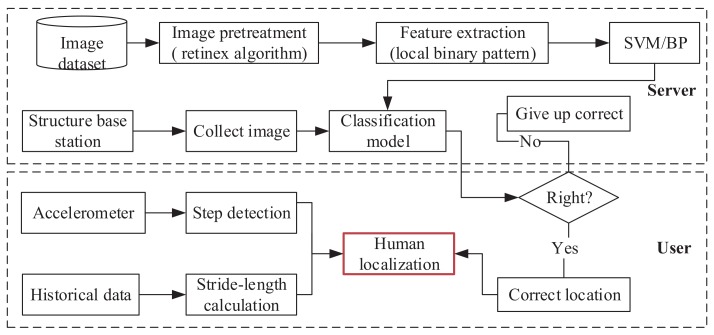
Algorithm architecture.

As shown in Figure 1, we use an asymmetric architecture for localization to simplify the calculation of the user’s side. The user only carries a simple device (accelerometer) to complete step detection. A server processes the computation for localization. Specifically, the localization procedure consists of the following steps:

- Step 1. Base station construction. The construction of base station consists of two phases (offline learning and online identification). The offline learning phase consists of the following steps: image pretreatment, feature extraction, and image classification by SVM [29] or BP [30]. The online identification phase is used in the classification model to identify different shapes for accurately locating users when they pass the base station.
- Step 2. Step detection. Utilize the accelerometer to complete step detection. First, the signal magnitudes are pre-processed by a smoothing filter. Then, the application of a low-pass filter can filter out high-frequency accelerations caused by user’s free movements, so as to better extract the low-band step component. Finally, a peak recognition algorithm is used to detect peaks and completes the step detection.
- Step 3. Stride-length calculation. Stride-length is different among various people. However, a person’s stride-length is almost unchanged in different cases. Thus, we can use the historical data to calculate stride-length. Specially, the historical data may have some outliers, those will be removed by the local outlier factor (LOF) [31] algorithm.
- Step 4. Real-time localization. The step and stride-length can be used to locate the user based on the coal mine map. Then, we can utilize the base station to correct the localization results when the user is passing by the base station.

For the rest of this section, we describe the technical contents of each step.

### 3.1. Base Station Construction

**Figure 2:**
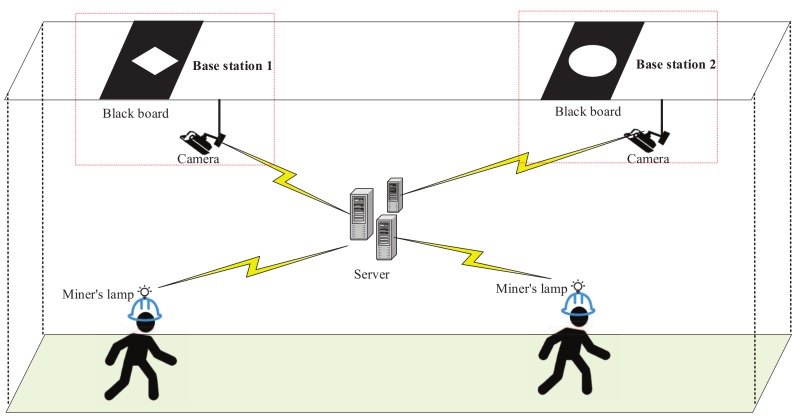
Base station.

To utilize the dark underground environment, we designed a low-cost base station with a camera. A base station consists of a miner’s lamp, a black board on the ceiling and a camera capturing the black board. Since every coal miner has a lamp, we simply transform it to irradiate unique shapes (such as triangle, rectangle, and circle) on the blackboard. These unique shapes can be seen as unique identifiers for every coal miner. Thus, coal miners can be accurately positioned when they pass by the base station by identifying the shape of the image captured by the camera. The sketch map of the base station is shown in Figure 2.

Given that every shape on the blackboard maps a coal miner, the key point of the base station is how to identify the different shapes. In this method, we used related image processing technology to identify various shapes. This method consists of three phases:

(1) Image pre-treatment: Some pictures may be blurred. To ensure the accuracy of image recognition, we pre-processed these pictures with an image enhancement operation based on retinex algorithm, which achieves the image enhancement operation by calculating the sensory response of lightness.

(2) Feature extraction: The local binary pattern (LBP) is the image feature and is a type of operator used to describe the local texture feature of an image [32]. It has many advantages, such as rotation invariance and gray invariance for image texture feature extraction. Given a pixel in the image, an LBP code is computed by comparing it with its neighbors:

(1) $$LBP_{P,R}=\sum_{p=0}^{P-1}s(g_{p}-g_{c})2^{p},s\left(x\right)=\left\{\begin{array}{cc} 1, & x\geq 0\\ 0, & x<0 \end{array}\right.$$

where $g_{c}$ is the gray value of the central pixel, $g_{p}$ is the value of its neighbors, *R* is the radius of the neighborhood and *P* is the total number of involved neighbors. Suppose that the coordinate of $g_{c}$ is (0,0), then the coordinates of $g_{p}$ are (Rcos(2πp/P),Rsin(2πp/P)). The gray values of neighbors that are not in the image grids can be estimated by interpolation. After the LBP of each pixel is identified, a histogram can be built to represent the feature of image.

(3) Training model: We can use the classical classification algorithm (SVM or BP) to train the classification model. SVM is a supervised learning model with associated learning algorithms that analyze data used for classification and regression analysis. Given a set of training examples, each marked as belonging to one or the other of two categories, an SVM training algorithm builds a model that assigns new examples to one category or the other, making it a non-probabilistic binary linear classifier [33,34,35]. BP is a method used in artificial neural networks to calculate a gradient that is needed in the calculation of the weights to be used in the network. It is shorthand for the backward propagation of errors, since an error is computed at the output and distributed backwards throughout the network layers [36,37,38,39]. In this paper we use a neural network with three hidden layer. Whether it is SVM or BP, the complexity of the training process is high, but we only need to use a trained model to identify. Only the complexity of the test process needs to be considered. The test complexity of these two algorithms is low and it only has one order of magnitude, which ensures the effectiveness of this algorithm.

### 3.2. Step Detection

Our system uses accelerometer readings to identify walking paces of the user. When the heel hits the ground, the accelerometer will exhibit the maximum amplitudes along all three axes [40]. Therefore, a peak recognition algorithm is devised to detect these hits, which in other words are steps. In the step detection, we only take the magnitude of the three-axis acceleration reading (i.e., $a=\sqrt{\left(a_{x}^{2}+a_{y}^{2}+a_{z}^{2}\right)}$) rather than accelerometer orientation into consideration.

First, the signal magnitudes are pre-processed by passing through a smoothing filter. Then, the application of a low-pass filter can filter out high-frequency accelerations caused by user’s free movements, so as to better extract the low-band step component which can be calculated online using the following first-order difference equation:

(2) $$a_{i}^{l}=a_{i-1}^{l}+\alpha \times \left(a_{i}-a_{i-1}^{l}\right).$$

where $a_{i}$ is the *i*th original acceleration magnitude and it becomes $a_{i}^{l}$ after passing through the low-pass filter. The α is set to 0.25 in default. After the low-pass filter, peaks in the filtered data can be detected by a peak recognition algorithm with a sliding window. Specifically, the one that is larger than all samples located in the range of $\left[t\left(i\right)-t_{w}/2,t\left(i\right)+t_{w}/2\right]$ will be recognized as a peak (i.e., a user step), and it is recorded as $a_{i}^{l}$. Considering that the user’s step frequency is in general lower than 3 Hz, the current implementation of the window size $t_{w}$ is set to 0.3 s.

### 3.3. Stride-Length Calculation

**Figure 3:**
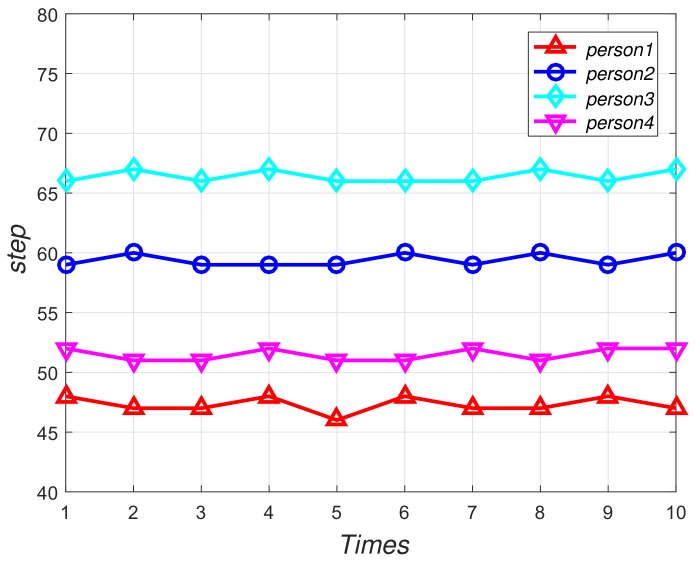
Preliminary experiment of stride-length.

We invited four volunteers to walk on the same aisle and record their steps respectively, and each person walks ten times. Figure 3 shows that each person has an essentially constant stride-length, where the *X*-axis means the times people walked and the *Y*-axis means the numbers of the steps these people walked in the same routine. Thus, the stride-length for each volunteer can be calculated by historical data which consist of the distance to adjacent base stations and users’ step information. We suppose that *N* pairs of adjacent base stations exist. The stride-length based on *i*th two adjacent base stations historical data can be calculated as follow:

(3) $$sl_{i}=\frac{d_{i}}{sp_{i}}.$$

where $d_{i}$ is the distance of ith two adjacent base stations and $sp_{i}$ is the user’s step between *i*th to adjacent base stations. By (3), we can obtain the *N* stride-length value. There may be outliers in these values. Thus, we use LOF algorithm to detect these outliers. On the basis of reference [31], the estimation of local density can be made by a specific distance where the sample point could be got through via its neighbors. Concretely, Specifically speaking, the definition of lrd(p) which means the local density of the sample point *p* is as follows:

(4) $$lrd\left(p\right)=1/\frac{\sum_{o\in k(p)}reach-disk(p,o)}{k}.$$

In the above equation, k(p) indicates the set of k-nearest neighbors of *p*, *o* represents any chosen point of k(p), the number of selected nearest neighbors is denoted by *k*, and the reachability distance is represented by reach−disk(p). The distance from target *p* to its *k* nearest neighbors is demonstrated by k−distance(p) and distance from *p* to *o* is d(p,o). Then the reach−disk(p) can be calculated as

(5) $$reach-disk_{k}\left(p,o\right)=\max\left(k-distance\left(p\right),d\left(p,o\right)\right).$$

The definition of LOF which means local outlier factor is the ratio of mean local densities of an object’s neighbors to the object’s local density. LOF of point *p* can be computed as follows:

(6) $$LOF\left(p\right)=\frac{\frac{1}{k}\sum_{o\in k(p)}lrd(o)}{lrd(p)}.$$

Every stride length is considered with single input of LOF function, the LOF values of *N* stride length is also calculated. The possibility of a point being an outlier is indicated by LOF. If the LOF value is approximately 1, the position of the point is within an area of homogeneous density, which means it is not an outlier. On condition that an outlier appears in a certain stride length, we believe that the stride length is an anomaly and we recommend removing it. After anomaly detection, we can get the final result by calculating the mean.

### 3.4. Real-Time Localization

A coal miner carrying an accelerometer can be located in real-time by related information (such as step, stride length, and underground map). As each intersection is set in a base station in the mine, the human localization is a linear localization. Thus, we can locate humans by step detection. Furthermore, to reduce the cumulative error, a large number of low-cost base stations is applied. When humans pass by the base station, we can correct their position based on the results of identifying shape. Although the image recognition results are highly accurate, some error still occurs. To improve the localization caused by image recognition error, we added an error control step before correcting the position. DE represents the distance error of the localization results of step detection and the base station. It can be calculated as follows:

(7) $$DE=\sqrt{{\left(x_{1}-x_{2}\right)}^{2}+{\left(y_{1}-y_{2}\right)}^{2}}.$$

where $\left(x_{1},y_{1}\right)$ is the location that is located by step and $\left(x_{2},y_{2}\right)$ is the location that is located by base station. If DE<m, then the identification results of the base station is right and we can have the correct location. By contrast, if DE≥m indicates that the identification results of the base station is false, and we cannot have the correct location.

## 4. Test-Bed Evaluation

### 4.1. Experiment 1: Indoor Environment

#### 4.1.1. Test-Bed Setup

**Figure 4:**
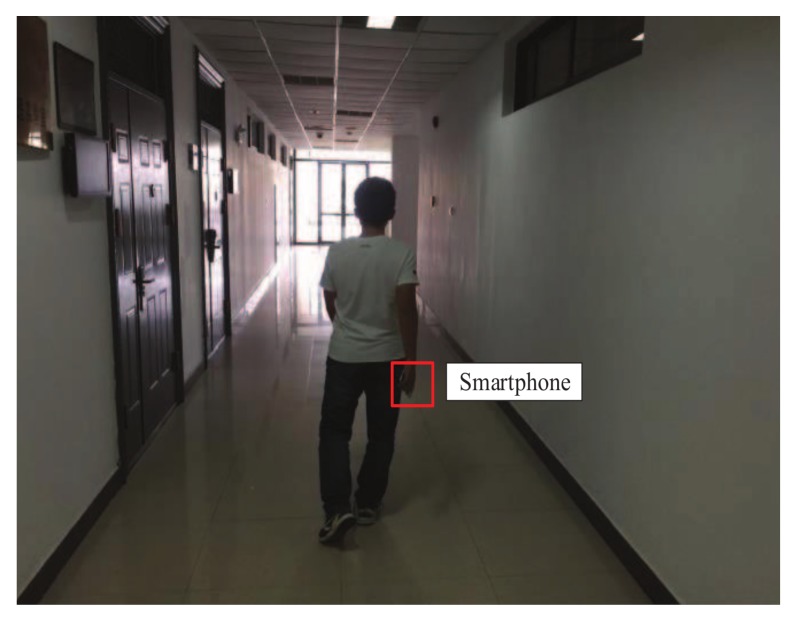
Indoor environment.

**Figure 5:**
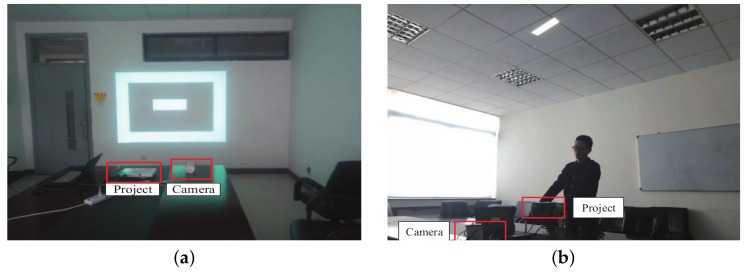
Analog base station. (**a**) static way; (**b**) dynamic way.

We simulated an underground roadway environment in the indoor environment as shown in Figure 4. We designed two cases to simulate real scenarios of moving people under coal mines. As shown in Figure 5a, analog base station with a Panasonic PT-SX1100 projection lamp which can irradiate unique shapes and a Hikvision DS-2CD1221-I3 camera were paced horizontally towards a blank wall, which was an ideal scene. Furthermore, Figure 5b presents a situation where the projector was erected up and rotated by certain angles, which more realistically conformed to the motion status of the person wearing the miner’s lamp. In addition, we made some noises by tuning on or off the indoor lanterns. In addition, four users take part in the evaluation of the performance of our algorithm, each user carry with a smartphone to record the step information. A new evaluation index is defined as $Accuracy=(N_{1}/N_{2})\times 100\%$, where $N_{1}$ means test samples recognized properly and $N_{2}$ represents all test samples.

#### 4.1.2. Base Station Performance

**Figure 6:**
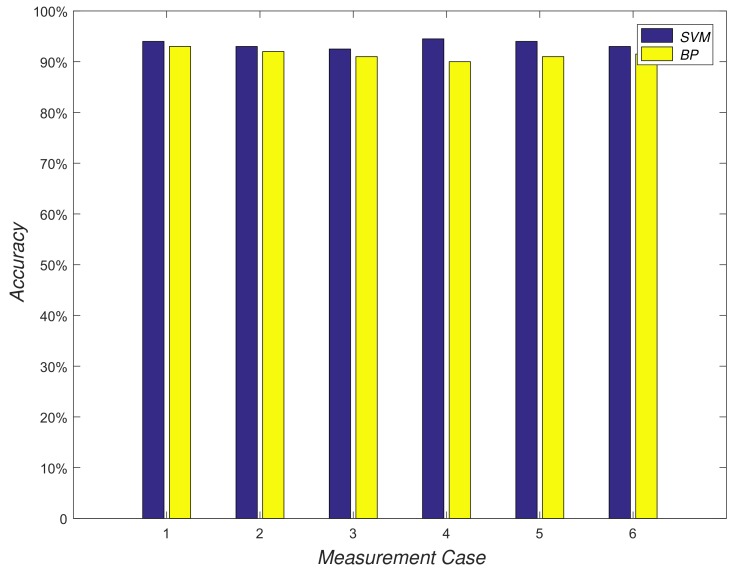
Accuracy of base station.

During this experiment, we unitized a projection lamp to produce different shapes on a black board and a camera was used to photograph these shapes. First, we collected 100 pictures that can be treated as training sets for every shape. Then, we unitized two classical classification algorithms (SVM and BP) to obtain two classification models with the training set. Next, we collected 50 images to test the classification model. In particular, to evaluate the robustness of this method, the images of the test set were collected in six different places. As shown in Figure 6, results verify that our method can accurately identify a variety of shapes where the accuracy of SVM and BP would be greater than 92% and 91% on average, respectively. Thus, we can claim that our base station has very good performance.

#### 4.1.3. Stride-Length Calculation Performance

**Figure 7:**
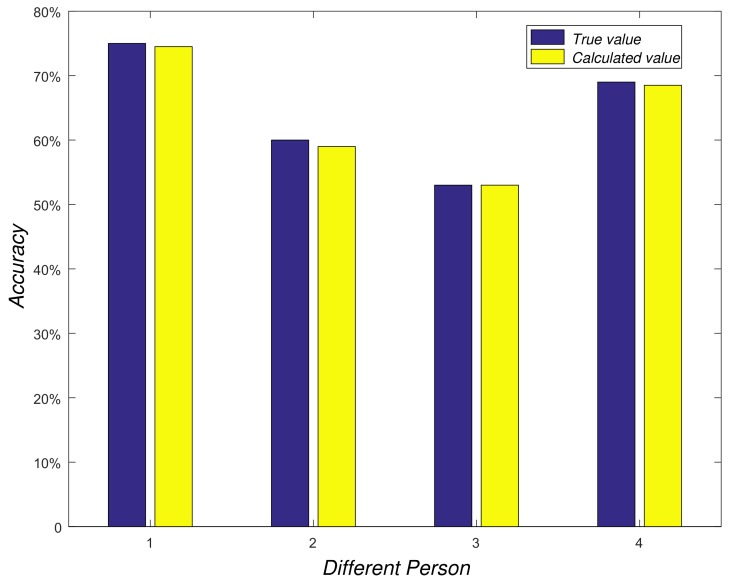
Calculating stride-length performance.

To evaluate the accuracy of the calculation of stride length, four users took part in our experiment. Each user walked freely in the corridor for a period of time and we recorded related data to calculate the stride length of each user. Then, we used the ruler to measure each user’s stride length as the true value. As shown in Figure 7, we can find that the calculated value is very close to the real value of each user.

#### 4.1.4. Step Detection Performance

**Figure 8:**
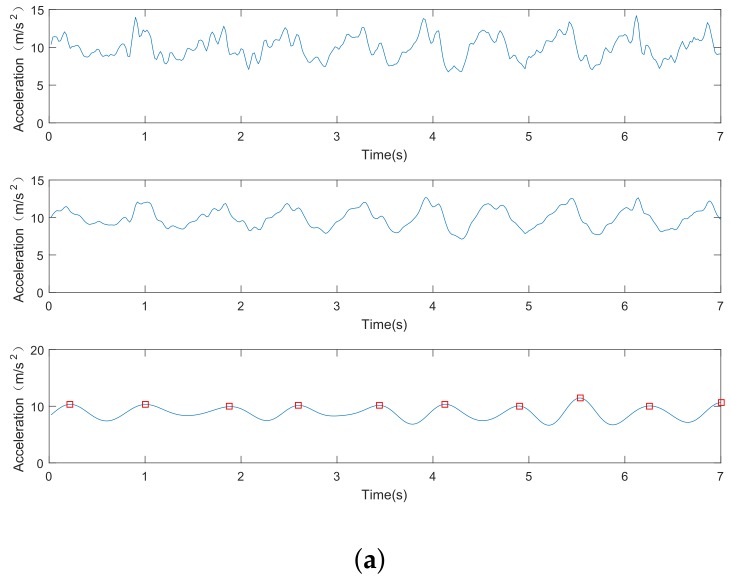

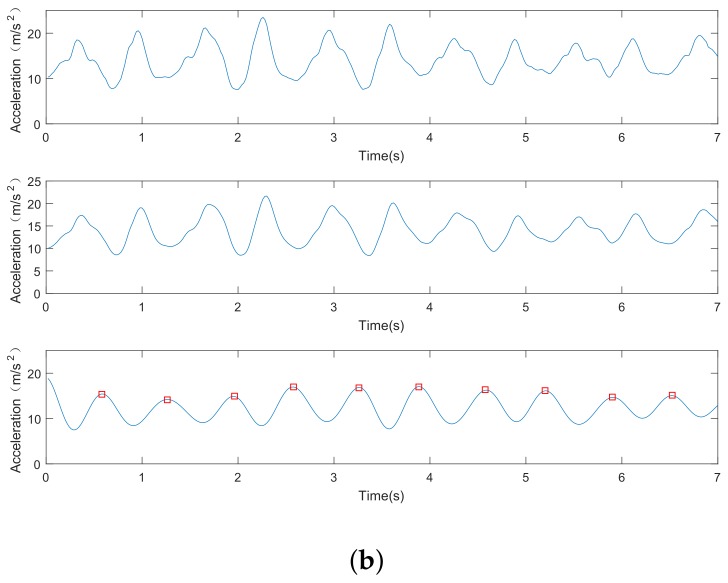
Data processing for step detection. (**a**) accelerometer in the hat; (**b**) accelerometer in hands.

**Figure 9:**
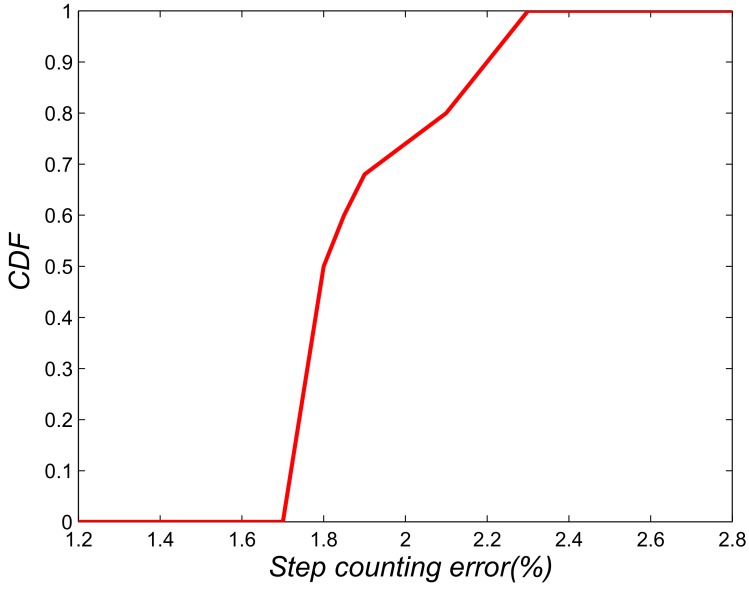
CDF of step detection.

Figure 8 shows the data processing of acceleration signals in step detection. For both Figure 8a,b, the original acceleration outputs from the accelerometer are displayed in the first row; the second and third rows show the smooth acceleration data and the corresponding low-band component, respectively. In addition, red squares highlight the recognized peaks in the third row. Figure 8b also shows the performances of our step detection algorithm even when the user swings his arm with the phone in hand. The step-counting errors are presented in Figure 9. As we can see, the error of the step detection method is below 2.3%, indicating a step count error of less than three steps per 100 steps.

#### 4.1.5. Localization Performance

**Figure 10:**
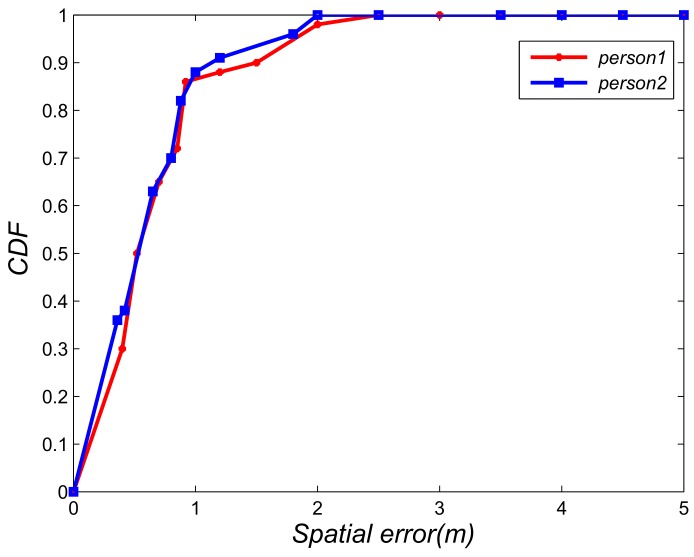
Localization result with base station distance is 30 m.

**Figure 11:**
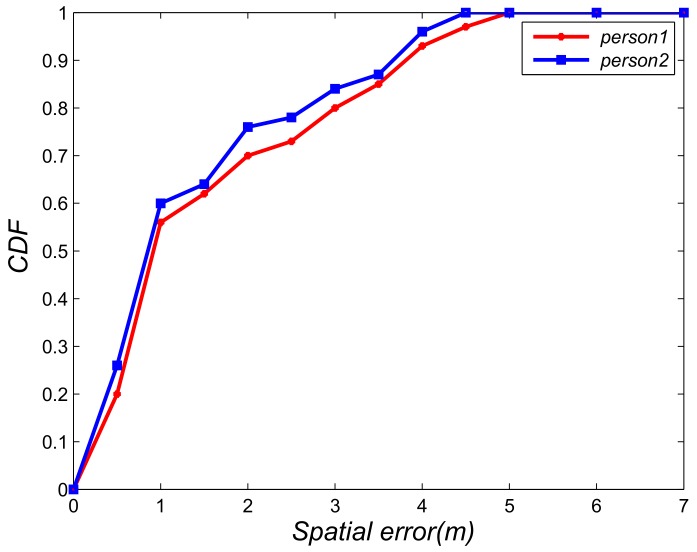
Localization result with base station distance is 50 m.

For our proposed algorithm, the distance between base stations will affect the localization. Given that our base station is low-cost, the stations are not far away. Thus, our proposed algorithm has good localization performance. We evaluated the localization performance of two different distances (50 and 100 m) of base stations. Figure 10 plots the spatial error of 50 m and the spatial errors are less than 2.1 m. Figure 11 plots the spatial error of 100 m and the spatial errors are less than 5.0 m.

### 4.2. Experiment 2: Underground Environment

**Figure 12:**
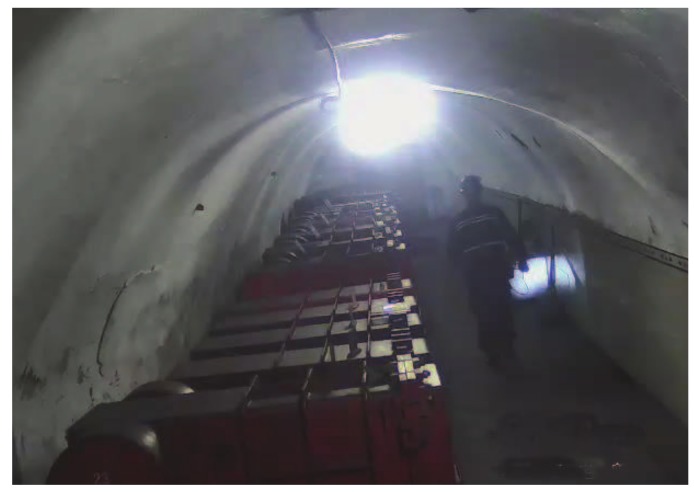
Underground environment.

**Figure 13:**
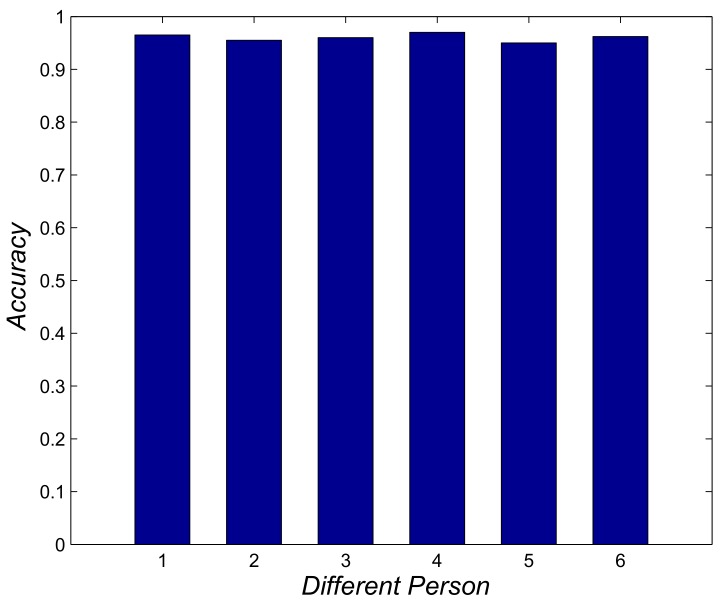
Localization result in underground environment.

We conducted a simple underground test to evaluate the performance of our proposed algorithm and the experimental scene, which is a 600-m underground tunnel, as shown in Figure 12. Six miners carrying with mobile device participated in our experiment. This experiment uses an off-line localization scheme. First, data are collected in an underground environment, and then these data are analyzed in the laboratory.The calculation results of the rate of spatial error during the localization process were 2.5 m or less. Meanwhile, the localization results are shown in Figure 13, where the average of 96% spatial error was 2.5 m or less for the six miners. Thus, our algorithm has a good localization performance in underground environments.

## 5. Conclusions

In this paper, we presented a special human localization algorithm for underground environments. This algorithm made full use of the special underground environment. A novel low-cost localization-based station was designed to solve the problem of the cumulative error of the step-based localization algorithm based on image recognition technologies. It should be noted in particular that the use of the system into underground coal mines requires the implementation of corresponding stringent safety directives/standards. Finally, we evaluated our algorithm in the indoor and underground environments. The results show that the base station identification accuracy was more than 94% and 96% of the spatial error were 2.5 m or less. At present, this work only designs the correction method of the base station for only one person. In future work, we aim to design an algorithm to solve multiple people simultaneously passing through the base station.

## References

[1] Yang Y., Li Y., Guo X. Underground personnel positioning sysyem based on low-power card reader. Proceedings of the International Conference on Automatic Control and Artificial Intelligence (ACAI 2012).

[2] Wei J., Sun S., Chen Y. An Improved TDOA Algorithm Applied Person Localization System in Coal Mine. Proceedings of the 2011 Third International Conference on Measuring Technology and Mechatronics Automation.

[3] Li F., Han P., Liu X. A Wireless Localization Method Used in Coal Mine. Proceedings of the 2007 2nd IEEE Conference on Industrial Electronics and Applications.

[4] Constandache I., Gaonkar S., Sayler M., Choudhury R.R., Cox L. EnLoc: Energy-Efficient Localization for Mobile Phones. Proceedings of the IEEE INFOCOM 2009.

[5] Scherhaufl M., Pichler M., Stelzer A. Localization of passive UHF RFID tags based on inverse synthetic apertures. Proceedings of the 2014 IEEE International Conference on RFID (IEEE RFID).

[6] Yang Z., Zhou Z., Liu Y. (2014). From RSSI to CSI: Indoor localization via channel response. ACM Comput. Surv..

[7] Xiao J., Wu K., Yi Y., Ni L.M. FIFS: Fine-Grained Indoor Fingerprinting System. Proceedings of the International Conference on Computer Communications and Networks (ICCCN 2012).

[8] Geng H.Q. (2008). The main environmental & social problems in China’s large coal mine construction and the countermeasures. J. China Coal Soc..

[9] Mukhopadhyay B., Sarangi S., Kar S. Performance evaluation of localization techniques in wireless sensor networks using RSSI and LQI. Proceedings of the 2015 Twenty First National Conference on Communications (NCC).

[10] Talvitie J., Renfors M., Lohan E.S. (2015). Distance-Based Interpolation and Extrapolation Methods for RSS-Based Localization with Indoor Wireless Signals. IEEE Trans. Veh. Technol..

[11] Guo Y., Huang K., Jiang N., Guo X. (2015). An Exponential-Rayleigh Model for RSS-Based Device-Free Localization and Tracking. IEEE Trans. Mob. Comput..

[12] Adib F., Katabi D. (2013). See through walls with WiFi!. Comput. Commun. Rev..

[13] Bahl P., Padmanabhan V.N. RADAR: An in-building RF-based user location and tracking system. Proceedings of the IEEE INFOCOM 2000, Nineteenth Joint Conference of the IEEE Computer and Communications Societies.

[14] Adib F., Kabelac Z., Katabi D. Multi-person localization via RF body reflections. Proceedings of the 12th USENIX Conference on Networked Systems Design and Implementation.

[15] Chen Y., Lymberopoulos D., Liu J., Priyantha B. FM-based indoor localization. Proceedings of the 10th International Conference on Mobile Systems, Applications, and Services.

[16] Hauschildt D., Kirchhof N. Advances in thermal infrared localization: Challenges and solutions. Proceedings of the 2010 International Conference on Indoor Positioning and Indoor Navigation.

[17] Kemper J., Hauschildt D. Passive infrared localization with a Probability Hypothesis Density filter. Proceedings of the 2010 7th Workshop on Positioning, Navigation and Communication.

[18] Fischer C., Muthukrishnan K., Hazas M., Gellersen H. Ultrasound-aided pedestrian dead reckoning for indoor navigation. Proceedings of the ACM International Workshop on Mobile Entity Localization and Tracking in Gps-Less Environments.

[19] Zhao Y., Liu Y., Ni L.M. (2013). VIRE: Virtual reference elimination for active RFID-based localization. Adhoc Sens. Wirel. Netw..

[20] Cangialosi A., Monaly J.E., Yang S.C. (2007). Leveraging RFID in hospitals: Patient life cycle and mobility perspectives. IEEE Commun. Mag..

[21] Prorok A., Tome P., Martinoli A. Accommodation of NLOS for ultra-wideband TDOA localization in single- and multi-robot systems. Proceedings of the 2011 International Conference on Indoor Positioning and Indoor Navigation.

[22] Fischer G., Klymenko O., Martynenko D., Luediger H. An impulse radio UWB transceiver with high-precision TOA measurement unit. Proceedings of the 2010 International Conference on Indoor Positioning and Indoor Navigation.

[23] Liu J.J., Phillips C., Daniilidis K. Video-based localization without 3D mapping for the visually impaired. Proceedings of the 2010 IEEE Computer Vision and Pattern Recognition Workshops.

[24] Qin Y., Wang F., Zhou C. (2015). A Distributed UWB-based Localization System in Underground Mines. J. Netw..

[25] Lourenço P., Guerreiro B.J., Batista P., Oliveira P., Silvestre C. (2016). Simultaneous localization and mapping for aerial vehicles: A 3-D sensor-based GAS filter. Auton. Robot..

[26] Shu Y., Bo C., Shen G., Zhao C., Li L., Zhao F. (2015). Magicol: Indoor Localization Using Pervasive Magnetic Field and Opportunistic WiFi Sensing. IEEE J. Sel. Areas Commun..

[27] Nerguizian C., Despins C., Affes S. (2006). Geolocation in mines with an impulse response fingerprinting technique and neural networks. IEEE Trans. Wirel. Commun..

[28] Chehri A., Fortier P., Tardif P.M. Application of Ad-hoc sensor networks for localization in underground mines. Proceedings of the 2006 IEEE Wireless and Microwave Technology Conference.

[29] Zhang Y., Li B., Lu H., Irie A., Xiang R. Sample-Specific SVM Learning for Person Re-identification. Proceedings of the 2016 IEEE Conference on Computer Vision and Pattern Recognition.

[30] Li C., Zong X., Gudake A Survey of Online Fault Diagnosis for PV Module Based on BP Neural Network. Proceedings of the 2016 International Conference on Smart City and Systems Engineering (ICSCSE).

[31] Breunig M.M., Kriegel H.P., Ng R.T., Sander J. LOF: Identifying density-based local outliers. Proceedings of the 2000 ACM SIGMOD International Conference on Management of Data.

[32] Guo Z., Zhang L., Zhang D. (2010). A Completed Modeling of Local Binary Pattern Operator for Texture Classification. IEEE Trans. Image Process..

[33] Zhang Y., Wang P., Ni T., Cheng P., Lei S. (2017). Wind Power Prediction Based on LS-SVM Model with Error Correction. Adv. Electr. Comput. Eng..

[34] Glowacz A. (2015). Recognition of acoustic signals of induction motor using fft, smofs-10 and LSVM. Eksploat. Niezawodn..

[35] Wilk-Kolodziejczyk D., Regulski K., Gumienny G. (2016). Comparative analysis of the properties of the nodular cast iron with carbides and the austempered ductile iron with use of the machine learning and the support vector machine. Int. J. Adv. Manuf. Technol..

[36] Glowacz A. (2018). Acoustic based fault diagnosis of three-phase induction motor. Appl. Acoust..

[37] Koprowski R., Lanza M., Irregolare C. (2016). Corneal power evaluation after myopic corneal refractive surgery using artificial neural networks. Biomed. Eng. Online.

[38] Tadeusiewicz R. (2015). Neural networks in mining sciences-general overview and some representative examples. Arch. Min. Sci..

[39] Gajewski J., Valis D. (2017). The determination of combustion engine condition and reliability using oil analysis by MLP and RBF neural networks. Tribol. Int..

[40] Zhou P., Li M., Shen G. Use it free: Instantly knowing your phone attitude. Proceedings of the 20th Annual International Conference on Mobile Computing and Networking.

